# The Role of Isotretinoin Therapy for Cushing's Disease: Results of a Prospective Study

**DOI:** 10.1155/2016/8173182

**Published:** 2016-02-29

**Authors:** Lucio Vilar, José Luciano Albuquerque, Ruy Lyra, Erik Trovão Diniz, Frederico Rangel Filho, Patrícia Gadelha, Ana Carolina Thé, George Robson Ibiapina, Barbara Sales Gomes, Vera Santos, Maíra Melo da Fonseca, Karoline Frasão Viana, Isis Gabriella Lopes, Douglas Araújo, Luciana Naves

**Affiliations:** ^1^Division of Endocrinology, Hospital das Clínicas, Pernambuco Federal University, Avenida Professor Moraes Rego, 1235, Cidade Universitária, 50670-901 Recife, PE, Brazil; ^2^Division of Endocrinology, Brasilia University Hospital, Brasilia, DF, Brazil

## Abstract

*Objective*. This prospective open trial aimed to evaluate the efficacy and safety of isotretinoin (13-*cis*-retinoic acid) in patients with Cushing's disease (CD).* Methods*. Sixteen patients with CD and persistent or recurrent hypercortisolism after transsphenoidal surgery were given isotretinoin orally for 6–12 months. The drug was started on 20 mg daily and the dosage was increased up to 80 mg daily if needed and tolerated. Clinical, biochemical, and hormonal parameters were evaluated at baseline and monthly for 6–12 months.* Results*. Of the 16 subjects, 4% (25%) persisted with normal urinary free cortisol (UFC) levels at the end of the study. UFC reductions of up to 52.1% were found in the rest. Only patients with UFC levels below 2.5-fold of the upper limit of normal achieved sustained UFC normalization. Improvements of clinical and biochemical parameters were also noted mostly in responsive patients. Typical isotretinoin side-effects were experienced by 7 patients (43.7%), though they were mild and mostly transient. We also observed that the combination of isotretinoin with cabergoline, in relatively low doses, may occasionally be more effective than either drug alone.* Conclusions*. Isotretinoin may be an effective and safe therapy for some CD patients, particularly those with mild hypercortisolism.

## 1. Introduction

Cushing's disease (CD), the most common form of endogenous Cushing's syndrome, is one of the most challenging endocrine disorders [1, 2]. It is mostly caused by an adrenocorticotropin- (ACTH-) secreting pituitary adenoma, leading to pathological hypercortisolism and, consequently, to increased morbidity and mortality particularly due to metabolic and cardiovascular complications [3–5].

Transsphenoidal surgery represents first-line therapy for most patients with CD but, even in expert hands, the remission rate of hypercortisolism is 65–90% in patients with microadenomas and only 43–60% in those with macroadenomas [6–9]. Moreover, recurrence of CD may occur in 15–66% of cases within 5–10 years of initially successful surgery [7–11], requiring further therapies [10, 12]. A second pituitary operation leads to remission in only 43–70% of patients and is associated with increased risk of hypopituitarism (41% to 50%) [4, 5, 12]. Pituitary irradiation and bilateral adrenalectomy are alternative treatment approaches [10, 12] but they can also be associated with serious complications, such as hypopituitarism [13] and Nelson's syndrome [14], respectively. Therefore, there has been growing interest in medical therapy for the management of CD, particularly in cases of persistent or recurrent hypercortisolism [4, 12, 15–18].

Worldwide, the most commonly prescribed drugs for CD have been ketoconazole (an adrenal steroidogenesis inhibitor), cabergoline (a D2-specific dopamine receptor agonist), and pasireotide (a multifunctional somatostatin receptor agonist) [4, 15–18]. However, their overall long-term efficacy in controlling hypercortisolism is low, ranging from 17 to 50% [19–26]. Better results have been observed when these medications are used in combination [20, 24, 27].

Novel agents that directly inhibit ACTH secretion by corticotrophs have been studied and, among them, retinoic acid, also called tretinoin, seems to be a promising approach [28]. In experimental studies, retinoic acid has been shown to decrease ACTH secretion both* in vitro* and* in vivo* through an action on proopiomelanocortin (POMC) gene transcription and to inhibit corticotropinoma development and proliferation [29]. Retinoic acid was also shown to be more effective than ketoconazole in dogs with CD, concerning hormonal control, tumor shrinkage, and survival time [30]. More recently, in a prospective study that involved only 7 patients with CD, UFC normalization was achieved by three of them within 180 days of treatment with retinoic acid [31].

The aim of this study was to evaluate the efficacy and safety of 13-*cis*-retinoic acid, also known as isotretinoin [32], in 16 CD patients with persistent or recurrent hypercortisolism after transsphenoidal surgery and to identify any possible predictor factors for a successful treatment.

## 2. Materials and Methods

### 2.1. Patients

Sixteen patients (nine men and seven women; mean age, 43.81 ± 8.10 years; age range 30–53) with persistent or recurrent CD after transsphenoidal surgery were included in this prospective open trial (Table 1). Subjects were selected from outpatients of the Division of Endocrinology at Hospital das Clínicas, Pernambuco Federal University, and Pernambuco Center for Diabetes and Endocrinology, Recife, Brazil.

The diagnosis of CD had been established following accepted criteria, that is, ACTH-dependent hypercortisolism associated with either (1) concordant ACTH increase by at least 50% of baseline in response to desmopressin, and serum cortisol decrease greater than 80% after an overnight high-dose (8 mg) oral dexamethasone suppression test, in the presence of an unequivocal pituitary lesion (greater than 6 mm) with MRI or (2) a central-to-periphery ACTH gradient higher than 2 (baseline) or 3 (after desmopressin) at inferior petrosal sinus sampling [3, 4, 33–38].

The diagnosis of the persistence or recurrence of CD after surgery was based on the detection of high UFC levels [3, 4]. Subjects previously submitted to radiotherapy were excluded from the study. In order to avoid the teratogenic potential of isotretinoin [32] and potential interferences of oral contraceptive drugs on cortisol measurements [39, 40], only men or postmenopausal women were recruited. Drugs that reduce cortisol levels (e.g., ketoconazole, cabergoline, and pasireotide) were withdrawn at least 2 months before the introduction of isotretinoin.

At the beginning of the study, all enrolled patients presented with high levels of UFC (range, 142.6–312.8 *μ*g/24 h (NR: 10–90 *μ*g/24 h); mean, 235.17 ± 41.07) and midnight salivary cortisol (range, 158.6-317.6 ng/dL (NR: up to 100 ng/dL); mean, 214.57 ± 44.25). Elevation of ACTH values was found in 9 (56.2%) patients (range, 41.7–89.4 pg/mL (NR: <46 pg/mL); mean, 60.18 ± 16.08) (Table 1).

Pituitary MRI depicted small residual tumors in eight of the 16 patients (50%) with a median diameter of 6.0 mm (range 4–9 mm) whereas in the remaining patients there was either no evidence of tumor or an empty sella (Table 1). Previous immunohistochemical studies showed in all tumors positive immunostaining for ACTH as well as low Ki-67 expression.

### 2.2. Study Protocol

The current study was mainly designed to evaluate the effectiveness of isotretinoin, a 13-*cis*-isomer of retinoic acid [32], in normalizing UFC levels in patients with persistent or recurrent Cushing's disease after pituitary surgery. The effect of the drug on midnight salivary cortisol (MSC) levels and ACTH levels was also assessed. Isotretinoin was administered orally at breakfast at the initial dose of 20 mg/day, which was progressively increased by 20 mg by every month, if needed and tolerated, until normalization of UFC levels or a maximal dose of 80 mg/day was reached.

Patients were classified as either responsive or nonresponsive if they reach or do not reach normalization of UFC levels, respectively. Patients who did not show decrease in UFC levels of at least 50% of pretreatment values nor UFC normalization at 6 months were withdrawn from the study and offered other treatment options. The remaining patients were maintained in the study for an additional 6 months (24 weeks).

Once monthly the patients were assessed clinically and by hormone and routine chemistry determinations. At every visit, the hormonal status was evaluated through the levels of UFC (measured in two consecutive days) and plasma ACTH and MSC. Blood chemistry (complete blood cells count, plasma blood glucose, lipids, transaminases, and serum creatinine) was assessed to search for possible side-effects of isotretinoin therapy, particularly leukocytosis, dyslipidemia, and impairment of liver and kidney functions [38, 41]. Clinical parameters evaluated included arterial blood pressure, anthropometric measurements, features of hypercortisolism, and possible isotretinoin induced side-effects.

The radiological study included the evaluation of the sellar region by MRI with axial, coronal, and sagittal slices in T1, before and after gadolinium, and in T2. The MRI was performed in each patient before the administration of retinoic acid.

The study was approved by the local ethics committee. All study participants provided written informed consent before enrollment had been reached.

### 2.3. Assays

Plasma ACTH was measured by a solid-phase two-site sequential chemiluminescent immunometric assay, whereas UFC levels were measured by a solid-phase competitive chemiluminescent enzyme immunoassay. Midnight salivary cortisol (MSC) levels were assessed by a high pressure liquid chromatography tandem mass spectrometry (HPLC-MS/MS) method. Normal ranges are 10–90 *μ*g/24 h for UFC, <46 pg/mL for plasma ACTH, and up to 100 ng/dL for MSC. Sensitivity of the methods was 5 pg/mL for ACTH, 0.20 *μ*g/dL for UFC, and 24 ng/dL for MSC. Intra-assay and interassay coefficients of variations were 5.0 and 9.0% for ACTH, 3.7 and 7.2% for UFC, and 5.6 and 7.9% for MSC, respectively.

### 2.4. Statistical Analysis

For comparison of categorical variables, the chi-squared test or the Fisher exact test was used where appropriate. Paired Student's* t-*test was performed for the comparative analysis of quantitative variables. Results are expressed as percentages or mean values ± SD, unless otherwise stated. A stratified analysis was performed aiming at finding potential associations among clinical, imaging, and biochemical characteristics with outcome.* P* values < 0.05 were considered statistically significant. STATA version 10.0 and SPSS version 16.0 were used as statistical software.

## 3. Results

### 3.1. Effect of Isotretinoin on Hormonal Levels

**Figure 1:**
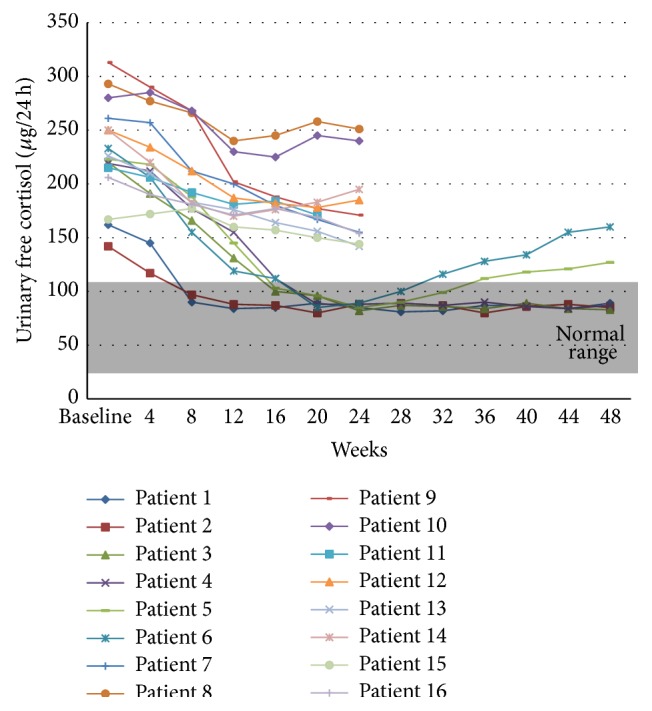
Patients UFC levels (baseline and during isotretinoin therapy). Ten patients (*7 to 16*) were withdrawn from the study at month 6 (week 24) due to poor response whereas the remaining 6 patients (*1 to 6*) were given an additional 6 months (24 weeks); 2 of them (*5 and 6*) subsequently developed relapse of hypercortisolism. Overall, 4 (25%) patients (*1 to 4*) achieved sustained UFC normalization.

Patients were treated for 6 to 12 months (mean, 8.25 ± 3.0; median, 6) at doses ranging from 60 to 80 mg daily (mean, 77.50 ± 6.83; median, 80). According to the study protocol, 10 patients were withdrawn from the study at 6 months (24 weeks) for not achieving UFC levels reduction of at least 50% of baseline values nor UFC normalization. The remaining subjects were treated for an additional 6 months. Normalization of UFC levels occurred in 6 patients (37.5%), but relapse of hypercortisolism was subsequently observed in two of them (Figure 1). Overall, four patients (25%) presented with UFC concentrations in the normal range at the end of the study after taking isotretinoin at a mean dose of 70 ± 11.54 mg/day (range, 60 to 80). Reductions in UFC levels ranging from 14.3 to 52.1% were found in the patients in whom sustained UFC normalization did not occur. All patients who normalized UFC excretion also reached normalization of MSC values. The response of ACTH levels did not necessarily match that of UFC and MSC values. Indeed, in responsive patients, ACTH levels decreased in the first 4 months of treatment but, in two of them, they later returned to the pretreatment range.

### 3.2. Predictors of Response to Isotretinoin

When responsive (*patients 1 to 4*) and nonresponsive patients (*patients 5 to 16*) were compared, no significant difference was found concerning gender and the proportion of patients with residual tumor on MRI (Table 2). By contrast, responsive patients had significantly lower mean age (38.5 ± 9.54* versus *45.58 ± 7.14; *P* < 0.01) as well as lower mean levels of UFC (183.98 ± 25.61* versus *239.09 ± 47.46 *μ*g/24 h; *P* < 0.01), MSC (202.33 ± 29.73* versus *243.37 ± 40.22 ng/dL; *P* < 0.01), and ACTH (54.02 ± 13.95* versus *62.23 ± 16.77 pg/mL; *P* < 0.01) (Table 2). Moreover, only patients with UFC levels below 2.5-fold of the upper limit of normal (ULN) achieved UFC normalization. Nevertheless, there was a high degree of overlapping in all these parameters. Likewise, 3 of the 12 nonresponsive subjects (25%) also presented with UFC values below 2.5-fold of the ULN (Table 1).

One of nonresponsive patients (*patient 5*) was subsequently given cabergoline in monotherapy but UFC normalization was not achieved at the maximal tolerated dose of 3 mg/week. Three months after the addition of isotretinoin (50 mg/day), UFC levels fell from 132 to 85.5 *μ*g/day. Of note, cabergoline dose could be later reduced to 2 mg/week. By contrast, this combined therapy failed to normalize UFC levels in* patient 6*.

### 3.3. Effect of Treatment on Clinical and Biochemical Features

Isotretinoin administration had also beneficial effects on clinical and biochemical features of hypercortisolism, which were much more pronounced among responsive patients. Indeed, in this group, there was a significant decrease in mean fasting plasma glucose, blood pressure, body weight, and waist circumference (Table 3). Mean reductions of body weight and waist circumference were of about 8%.

### 3.4. Isotretinoin Safety

The drug was on average well tolerated except for mild and mostly transient conjunctival irritation, cheilitis, mucositis, nausea, headache, and arthralgias. Overall, 7/16 patients (43.7%) experienced clinical side-effects and 5 (31.2%) developed abnormal laboratory parameters, mainly represented by mild elevations of triglycerides and transaminases concentrations (less than twice ULN). Clinical side-effects could be controlled by patients avoiding sun exposure as far as possible and applying moisturising cream to inflamed portions of their skin and to their lips.

## 4. Discussion

Unlike prolactinomas and acromegaly, currently there is not a very effective medication that directly inhibits ACTH secretion by the corticotroph tumor [1, 2]. Worldwide, the most widely prescribed drug for the treatment of Cushing's syndrome has been ketoconazole, an antifungal agent that blocks several steps of adrenal steroidogenesis [4, 11, 15]. However, in the largest series to date (*n* = 200), normalization of UFC levels was found in only 49% of patients at the last follow-up [19].

Currently available neuromodulators of ACTH release showing efficacy in patients with CD include cabergoline and pasireotide [20, 24–28]. The rationale for the use of cabergoline in CD was the demonstration that the dopamine receptor subtype 2 (D2) is expressed in approximately 80% of human corticotroph adenomas and that these adenomas can be responsive to the ACTH-inhibiting actions of D2-agonists* in vitro* [42, 43]. In four clinical studies totalizing 80 CD patients who have failed surgery, long-term treatment with cabergoline at a dose of 1–7 mg weekly resulted in control of hypercortisolaemia in 22–50% of patients [20–23]. Remission has also been reported as primary therapy [44]. Nevertheless, approximately 25% of patients responsive to cabergoline experience escape phenomenon at 2–5 years [16]. Better efficacy has been reported by the combination of cabergoline with ketoconazole [27]. Indeed, in two small series involving 26 patients with CD, the success rate of combination therapy ranged from 67% to 79% and no treatment escape was reported [20, 45].

Pasireotide is a novel somatostatin analogue with high affinity for receptor subtypes 1, 2, 3, and particularly 5 (SSTR5), which was shown to be strongly overexpressed in corticotroph adenoma cells [46, 47]. By contrast, octreotide and lanreotide bind mainly to STTR2 whose expression in corticotropinomas is low due to its downregulation by hypercortisolism [46, 47]. In three recent studies, the rate of response (defined as normalization of UFC levels) ranged from 17% to 29% [24–26]. However, it was much higher (50%) in patients with mild CD (UFC 1.5–2-fold above ULN) who were given 900 *μ*g twice daily [25]. The major disadvantage of pasireotide therapy is hyperglycemia which can develop or get worse in up to 73% of patients [16, 25, 28].

There is also increasing evidence that retinoic acid may be a potentially useful novel therapy for CD [29–31, 48]. The rationale for this approach is the fact that the mode of action of retinoic acid involves an interaction with retinoic acid receptors (RAR) and retinoid X receptors (RXR) which are often found in corticotropinomas and other pituitary adenomas [49]. Isotretinoin, a 13-*cis*-isomer of retinoic acid, has a low affinity for RAR and RXR, but it may act as a prodrug that is converted intracellularly to metabolites that are agonists for RAR and RXR nuclear receptors [50–52]. Isotretinoin has at least five biologically important metabolites: 13-*cis*-4-*oxo*-retinoic acid (4-*oxo*-isotretinoin), all-*trans*-retinoic acid (tretinoin), all-*trans*-4-*oxo*-retinoic acid (4-*oxo*-tretinoin), 9-*cis-*retinoic acid, and 9-*cis*-4-*oxo*-retinoic acid [52].

In experimental studies involving AtT-20 pituitary ACTH-secreting tumor cells, retinoic acid was shown to reduce ACTH secretion* in vitro* by inhibiting the transcriptional activity of the transcription factors AP1 and Nur on the POMC gene, which encodes ACTH [29, 48]. It was also shown that retinoic acid inhibits cell proliferation and induces apoptosis in ACTH-secreting tumor cells [29, 48]. This antiproliferative effect, further confirmed in mice implanted with corticotroph tumors [29], seems to be mediated by bone morphogenetic protein-4 (BMP-4) whose expression is induced by retinoic acid [53].

It is noteworthy that the inhibitory action of retinoic acid seems to be restricted to ACTH-secreting tumor cells, since, in rat normal pituitary cells, neither ACTH, prolactin, nor growth hormone is affected by the treatment [48]. This finding is thought to be related to inhibition of retinoic acid response pathways by COUP-TF1 (chicken ovalbumin upstream promoter transcription) [29, 54, 55]. The latter, an orphan receptor that belongs to the steroid/thyroid hormone receptor superfamily, is usually expressed in normal corticotrophs but not in ACTH-secreting tumors [48, 54]. The apparent selectivity of retinoic acid for tumoral corticotrophs undeniably would be an additional benefit of the treatment.

Retinoic acid also acts directly on the adrenal cortex once it was shown to inhibit adrenal cortex cell proliferation and forskolin-stimulated corticosterone secretion cells [29, 48]. Moreover, ACTH inhibition was also observed in tumor cells with lung origin, demonstrating that the ACTH biosynthesis is affected by retinoic acid in different tumor types cells [48]. Altogether, these findings could also suggest a potential therapeutic role of the drug in other forms of endogenous Cushing's syndrome.

In the first clinical trial, published in 2006, dogs with Cushing's disease were given retinoic acid (*n* = 22) or ketoconazole (*n* = 20) for 6 months and treatment outcomes were very promising [30]. In fact, retinoic acid therapy reduced ACTH and cortisol secretion, improved clinical manifestations of hypercortisolism, and, unlike ketoconazole, induced shrinkage of the pituitary tumor. Moreover, the survival time after initiation of treatment was significantly longer in the retinoid acid group compared with the ketoconazole group [30].

More recently, Giraldi et al. [31] evaluated the efficacy of long-term treatment with retinoic acid in 7 patients with CD. Overall, five of seven patients (71.4%) exhibited a clear-cut decrease (at least 50%) in UFC excretion that led to normalization in three of them (42.8%). In addition, clinical features of hypercortisolism, particularly glycemic control and body weight, were ameliorated. After cessation of retinoic acid administration, UFC appeared to slowly rebound to pretreatment levels. However, very interestingly, long-lasting normalization of UFC was observed in one patient after drug discontinuation, suggesting a durable inhibitory effect of retinoic acid on the tumoral corticotroph [31], similarly to what happens in some prolactinomas patients after cabergoline withdrawal [56, 57] and a few acromegalic subjects following octreotide LAR interruption [58, 59].

In our study, we demonstrated in a larger number of patients (*n* = 16) that the therapy with isotretinoin, in a median dose of 80 mg/day (range, 60 to 80) for 6 to 12 months, yielded normalization of UFC and midnight salivary cortisol (MSC) in 6 subjects (37.5%), but relapse of hypercortisolism subsequently occurred in two of them. UFC reductions ranging from 14.3% to 55% were found in nonresponsive subjects. Though analysis is limited by small numbers overall in each group, responsive patients, as compared to the nonresponsive ones, presented with significantly lower mean age as well as significantly lower mean levels of ACTH, UFC, and MSC. However, there was a high degree of overlapping in all these parameters in both groups. Of note, only patients with UFC levels below 2.5-fold ULN achieved UFC normalization. Nevertheless, this finding was also observed in 25% of nonresponsive patients. In the above-mentioned study predictor factors for a successful response to retinoic acid were not evaluated [31].

Isotretinoin was on average well tolerated and none of our patients had to interrupt the treatment. However, the typical side-effects of the drug (e.g., conjunctival irritation, cheilitis, nausea, headache, and arthralgias) were experienced by ~44% of patients, although they were mild and mostly transient. Similar findings were reported by Giraldi et al. [31].

As shown in Table 4, a combined analysis of our data with those of the Italian study [31] would indicate that about 30% of patients with CD could benefit from the therapy with retinoic acid or 13-*cis*-retinoic acid. Some factors could explain the lack of efficacy of this approach in most patients, such as the known variable expression of the retinoid X receptors in ACTH-secreting adenomas [29], as well as the occasional expression of COUP-TF1 in these tumors [30, 31], which could antagonize the therapeutic effect of both retinoids.

The combination therapy with retinoic acid or isotretinoin and cabergoline also seems to be attractive as it could enable the use of lower doses and hence better treatment safety and tolerability. Moreover, as demonstrated in a recent* in vitro* study, 9-*cis*-retinoic acid induced a functional dopamine receptor type 2 (DRD2) in the pituitary corticotroph cell line AtT20 and increased cell sensitivity to the dopamine agonist bromocriptine (BCR) via a mechanism only partially related to corticotroph-to-melanotroph transdifferentiation [60]. In addition, in nearly 45% of corticotropinoma-derived primary cultures, the combined administration of 9-*cis*-retinoic acid and bromocriptine lowered the steady-state level of the ACTH precursor proopiomelanocortin (POMC) more efficiently than either of the drugs alone [60]. Accordingly, in one of our nonresponsive patients, cabergoline therapy, at the maximal tolerated dose of 3 mg/week, failed to normalize UFC levels, which was achieved after the addition of 50 mg/day of isotretinoin. It is noteworthy that cabergoline dose could be subsequently reduced to 2 mg/week.

## 5. Conclusion

The findings of the current study demonstrated that isotretinoin may be an effective therapy for some patients with Cushing's disease, particularly those with mild hypercortisolism. In our series, 25% of patients achieved sustained UFC normalization, all of them with UFC levels below 2.5-fold ULN.

## Figures and Tables

**Table 1:** Characteristics of patients.

Patients	Age (years)	Gender	BaselineUFC (*μ*g/24 h)	Last UFC (*μ*g/24 h)	Baseline ACTH (pg/mL)	Last ACTH (pg/mL)	Baseline MSC (ng/dL)	Last MSC (ng/dL)	Isotretinoin dose (mg/d)	Treatment duration (months)	MRI findings
1	30	Male	168	89.5	65.6	45.4	174	96.6	60	12	SRT
2	34	Male	142.6	85.5	41.7	41.2	158.6	92.5	60	12	ES
3	38	Male	220	83.3	42.2	44.3	174.6	90.4	60	12	SRT
4	52	Female	219	87.7	66.6	44.1	166.7	97.4	80	12	NVT
5	53	Female	223.4	127	79.4	49.7	212.4	133.8	80	12	SRT
6	45	Male	261.3	160.7	44.6	49.9	230	170	80	12	ES
7	44	Male	233.7	112	45.4	48.8	166.4	139	80	6	ES
8	39	Male	293.5	251.5	69.3	69.5	188	121	80	6	NVT
9	41	Male	312.8	171.5	74.4	77.7	272	199.4	80	6	SRT
10	40	Male	280	239.9	77.2	71.4	250	200	80	6	SRT
11	48	Female	215.5	165.2	45.8	43.4	317.6	214.3	80	6	NVT
12	50	Female	250.3	185	42.8	41.7	255	130.7	80	6	NVT
13	51	Female	236	142.3	44.4	43.2	210	154	80	6	NVT
14	53	Female	250.22	195.6	89.4	77.8	238.4	212.6	80	6	SRT
15	53	Female	187.4	144	63	54.4	210.4	178.4	80	6	SRT
16	30	Male	206.4	154	71.1	62.2	209	172	80	6	SRT

Responsive patients, 1 to 4; nonresponsive patients, 5 to 16.

UFC, urinary free cortisol; MSC, midnight salivary cortisol; SRT, small residual tumor; ES, empty sella; and NVT, no visible tumor.

Normal ranges: UFC, 10–90 *μ*g/24 h; ACTH, <46 pg/mL; and MSC, up to 100 ng/dL.

**Table 2:** Comparison of responsive and nonresponsive patients.

Features	Responsive patients (*n* = 4)	Nonresponsive patients (*n* = 12)	*P *value
Mean age (years)	38.5 ± 9.54	45.58 ± 7.14	<0.01
Mean UFC (mg/day)	202.33 ± 29.73	243.37 ± 40.22	<0.01
Mean ACTH (pg/mL)	54.02 ± 13.95	62.23 ± 16.77	<0.01
Mean midnight salivary cortisol (ng/dL)	168.47 ± 7.49	229.93 ± 40.31	<0.01
Mean retinoic acid dose (mg/day)	70.0 ± 11.54	80.00 ± 0.00	0.02
Rate of visible tumor on MRI (%)	50	50	1
Rate of male patients (%)	75	50	0.58
Rate of female patients (%)	25	50	0.58
Rate of patients with UFC levels ≤ 2.5-fold ULN (%)	100	25	0.02

**Table 3:** Effect of isotretinoin (13-*cis*-retinoic acid) on clinical and biochemical parameters in responsive patients.

Parameter	Baseline	At the end of the study	*P* value
Mean HbA1c (%)	7.60 ± 0.10	7.06 ± 0.15	0.50
Mean fasting plasma glucose (mg/dL)	169.33 ± 7.23	123.66 ± 5.13	0.01
Mean weight (kg)	80.5 ± 4.85	73.77 ± 4.01	0.01
Mean waist circumference (cm)	93.37 ± 6.26	85.02 ± 4.78	0.01
Mean systolic blood pressure (mmHg)	152.5 ± 23.27	120.0 ± 1.63	0.01
Mean diastolic blood pressure (mmHg)	111.25 ± 13.14	83.75 ± 4.78	0.01

**Table 4:** Efficacy of retinoic acid (RA) and 13-*cis-*retinoic acid (cRA) in normalizing UFC levels in patients with Cushing's disease (*data from 2 studies*).

Authors (year) [ref.]	Number of patients	Drug	Dose (mg/day)	Treatment duration (months)	Rate of UFC normalization (%)
Giraldi et al. (2012) [31]	7	RA	80	6–12	42.8

Current study	16	cRA	Range, 60–80 Median, 80	6–12	25.0

All	23	—	Range, 60–80	6–12	30.4
